# Whetting disadvantaged adults’ appetite for nutrition education

**DOI:** 10.1017/S1368980016002512

**Published:** 2016-09-19

**Authors:** Simone Pettigrew, Nicole Biagioni, Sarah Moore, Iain S Pratt

**Affiliations:** 1 School of Psychology and Speech Pathology, Curtin University, GPO Box U1987, Perth, WA 6845, Australia; 2 Cancer Council Western Australia, Perth, WA, Australia

**Keywords:** Nutrition education, Adults, Programme evaluation, Qualitative

## Abstract

**Objective:**

To identify the features of a nutrition education programme for disadvantaged adults deemed most attractive and useful by participants.

**Design:**

A two-year, multi-method, qualitative evaluation of pre and post data collected from programme participants. Data were imported into NVivo10 for coding to facilitate a thematic analysis.

**Setting:**

Western Australia.

**Participants:**

Individuals attending the Western Australian FOODcents nutrition education programme that is designed to provide knowledge and skills needed to consume a healthy diet on a budget. Focus groups were conducted several weeks after course completion (five groups, forty-seven participants), observations were conducted during FOODcents sessions (thirty-one observation episodes, 237 participants), and open-ended questions were asked in pre–post hard-copy surveys administered in sessions (*n* 927) and an online survey administered on average six weeks after course completion (*n* 114).

**Results:**

The course attributes that were found to be especially important to participants were: (i) user-friendly, practical information that could be immediately translated to their daily lives; (ii) experiential learning that involved direct contact with food products; and (iii) opportunities for social interaction. These aspects of nutrition education were described as being highly influential in the decision to participate in the course, the application of the information in their subsequent food purchase and preparation activities, and their word-of-mouth communications with others about the course.

**Conclusions:**

Incorporating aspects of most importance to participants into nutrition education programme delivery and promotion may increase joining rates, enjoyment, satisfaction with course content and, ultimately, the uptake of recommended behaviours.

Adult nutrition education has an important role to play in improving nutrition-related attitudes, knowledge and behaviours as part of a comprehensive approach to population health^(^ ^1^ ^)^. Examples of established adult nutrition education programmes include the Expanded Food and Nutrition Education Program in the USA^(^ ^2^ ^,^ ^3^ ^)^ and FOODcents in Australia^(^ ^4^ ^,^ ^5^ ^)^. These programmes aim to provide disadvantaged individuals with the information and skills they need to improve their diets within their limited budgets. In doing so, they have the potential to reduce existing health inequalities resulting from differences in socio-economic status^(^ ^6^ ^,^ ^7^ ^)^.

Continuing investment in adult nutrition education is contingent upon the ability to demonstrate programme effectiveness, making rigorous evaluations an important aspect of programme implementation^(^ ^8^ ^–^ ^10^ ^)^. As a result, research to date has focused on assessing the extent to which programmes are able to achieve knowledge and behavioural outcomes^(^ ^2^ ^–^ ^4^ ^,^ ^6^ ^,^ ^11^ ^,^ ^12^ ^)^. To produce generalisable results, these studies have typically been quantitative in design, thereby relying on participants being adequately literate to complete surveys. The perspectives of the most disadvantaged participants are inevitably missing when such methods are employed.

Given the voluntary nature of adult nutrition education, understanding the extent to which programmes meet participants’ self-identified needs is important to facilitate optimal levels of recruitment and retention^(^ ^13^ ^)^. The general adult education literature offers insights of relevance to this issue. It is noted that adult education needs to be understood as both a cognitive and affective activity, and both aspects need to be accommodated to optimise programme outcomes^(^ ^14^ ^,^ ^15^ ^)^. Behavioural change resulting from exposure to education is more likely to occur when programme participants are actively engaged in the learning process and experience satisfying encounters with others during the learning experience^(^ ^16^ ^)^. It is also important for participants to be able to choose the topics to be covered, ask questions throughout the learning process, and interact with new information at the motor learning (physical) level so they can ‘feel’ rather than just read or hear the information^(^ ^15^ ^–^ ^17^ ^)^.

The aim of the present study was to identify the programme features deemed most attractive and useful by individuals attending the Australian FOODcents nutrition education and food budgeting programme. FOODcents has been providing disadvantaged Western Australians with practical advice on healthy eating on a budget since 1992^(^ ^5^ ^,^ ^18^ ^)^. Participants are recruited through a range of social service and community agencies, and include recent immigrants, those with physical and mental disabilities, the elderly and Aboriginal Australians. In most instances agencies submit requests for courses to be delivered to groups of their clients, although individuals can also register to attend a course. Most courses are delivered to small groups, typically comprising five to fifteen people, in community settings such as recreation centres, community halls and agency offices. The groups are often pre-existing, such as mothers’ groups, church groups and immigrant groups. Programme delivery is via face-to-face sessions involving a combination of information presentation, skills training and cooking classes. The courses are delivered by facilitators with tertiary nutrition training and translators are used to assist in providing instruction where the courses are delivered to non-English-speaking groups.

Each FOODcents course can range from one to eight sessions in duration, depending on the needs and preferences of participants. Similarly, participants can nominate the nutrition-related concepts and skills of most interest and relevance to them, and the courses are delivered according to these preferences (for further information relating to programme content, see Pettigrew *et al*.^(^ ^4^ ^)^). Previous FOODcents evaluations have utilised pre–post surveys to assess knowledge and behaviour change, and have demonstrated that among those able to complete the surveys there were significant improvements in all outcome variables^(^ ^4^ ^,^ ^6^ ^)^. The present study extends these evaluations by incorporating a substantial qualitative data collection component to access a broader range of disadvantaged participants, including those who are unable to respond to written surveys.

## Method

Novel and comprehensive data approaches are required to access low-literate individuals participating in nutrition education programmes because of the difficulties they can experience in engaging with the research process^(^ ^19^ ^)^. To address this need, the present study included a range of data collection methods that catered to diverse reading and writing abilities. In particular, participant observation episodes were undertaken during thirty-one FOODcents sessions (237 participants) to allow engagement levels to be assessed. These observation episodes involved independent research assistants physically attending sessions to document participant interactions (e.g. questions asked and engagement with the materials, activities and instructors) and to make conversation with participants at the end of the session to assess their perceptions of their FOODcents experience. Prior to the commencement of the observation episodes, the FOODcents educator (or translator) announced the presence of the researcher and explained the purpose of the study. Attendees were advised that the researcher would be available to talk to at the end of the session, but that participation in the evaluation was entirely voluntary. In the case of immigrant participants, translators were present to relay the course content and to assist with the conversations with the researcher.

The second data collection method was focus groups (five groups, forty-seven participants), which were moderated by an experienced qualitative researcher (the first author) and conducted with groups of particular interest, including Aboriginal Australians, unemployed young adults and mothers of young children. Focus groups were especially appropriate for use with these participants because the courses were delivered in contexts where these individuals meet as pre-formed groups (e.g. an Aboriginal community centre and a skills-building centre for unemployed young people) and hence the participants were available for subsequent evaluation sessions at the same time and place at a later date. In addition, familiarity with the location and peer group facilitated active discussion and interaction during these focus group sessions.

To supplement the qualitative data obtained via the observation episodes and focus groups and to provide additional quantitative data to be used in other analyses^(^ ^4^ ^,^ ^6^ ^)^, in-session (*n* 927) and online (*n* 114) surveys were administered as part of the broader evaluation (see Pettigrew *et al*.^(^ ^4^ ^)^ for survey details). The in-session surveys comprised hard-copy surveys that were completed at the end of the last course session. The online survey was administered on average six weeks after course attendance to assess the extent to which participants were utilising their new nutrition knowledge. There was some participant cross-over, with the online survey sample being comprised of those who had completed an in-session survey and provided an email address for follow-up contact.

Over the evaluation period, 3857 individuals attended a FOODcents course, indicating an inclusion rate of 31 %. Evaluations were not conducted in courses that were short in duration (e.g. one-hour sessions on food labelling) because the time taken to survey or interview participants would consume a relatively large proportion of the course time. In addition, groups that were considered too vulnerable, such as people with severe mental illness or in extreme poverty, were not included in the evaluation to minimise the effort required of participants.

Open-ended questions in the in-session and online surveys related to participants’ motivations to attend FOODcents, their favourite and least favourite aspects of the course, and the elements of the experience they found most useful and memorable. The survey respondents were also given the opportunity to provide general feedback on any issue they considered relevant. This form of data collection provided a cost-effective means of allowing those participants with higher literacy levels to provide as much feedback as they wished and to make suggestions for programme improvement.

Detailed demographic data were captured for those responding to the in-session and online surveys, but this was not possible for the focus groups and observation episodes, especially where translators were used to relay questions and answers and the interactions therefore needed to be kept brief. In these instances, just participant gender was recorded. Overall, about three-quarters of the participants involved in the evaluation were female. Participants were aged from young adults to the elderly, and just over half of the survey respondents reported having children under 18 years of age.

Data collection occurred over a period of two years to ensure access to a broad range of participants attending the full spectrum of FOODcents courses (i.e. courses of varying duration and covering different nutrition education topics). Ethics approval for the study was obtained from the Curtin University Human Research Ethics Committee. Survey respondents and focus group participants provided written consent, and participants involved in the observations provided verbal consent before participating in conversations. With the permission of the participants, the focus groups were audio-recorded and subsequently transcribed verbatim.

All qualitative data were imported into NVivo10 qualitative data analysis software (QSR International) for coding and analysis. The overall approach to analysis was inductive^(^ ^20^ ^)^, with emergent coding conducted by the first author. Consistent with an inductive approach, the constant comparative method was used to examine similarities and differences within individual participants’ responses and across the responses of different individuals^(^ ^21^ ^)^. The quality criteria of prolonged engagement, source triangulation and peer debriefings among members of the author team were used to enhance the trustworthiness of the findings^(^ ^22^ ^)^.

The primary themes and sub-themes that were generated from the data are presented as a conceptual framework in Table 1 and described in more detail below. Quotes from participants are provided, with relevant demographic details listed where available. The selected quotes exemplify common viewpoints presented across the sample while ensuring representation of the wide range of participants involved in the evaluation study.

**Table 1:** Framework of Western Australian participants’ preferred attributes of an adult nutrition education programme

Desired functional outcomes	Experiential aspects	Social outcomes
Knowing what to buy	Cooking	Social interaction
∙ For health ∙ For budget		
Knowing how to buy	Learning activities	Benefits for others
∙ Labels ∙ Healthy eating pyramid ∙ Mnemonics ∙ In bulk		∙ Children ∙ Grandchildren ∙ Other family members ∙ Friends
Knowing how to prepare	Interactive learning	Word-of-mouth communications
∙ Substitutes ∙ Hygiene		

## Results

Overall, participants expressed high levels of enjoyment of and satisfaction with the course, which was equally apparent between different gender and age groups and between those participating in the various forms of data collection. The consistency in outcomes across the sample also provided assurance of data saturation due to the identified themes being fully explicated both within and across data sources^(^ ^21^ ^)^. The dominant sentiments expressed by participants related to their appreciation for their enhanced ability to select and prepare healthy foods in a manner that would benefit both themselves and their loved ones. Also important was the experiential aspect of learning, specifically the social context and the pedagogical approaches adopted. These outcomes are outlined below under the themes of ‘Desired functional outcomes’, ‘Experiential aspects’ and ‘Social context’.

### Desired functional outcomes

The participants appeared highly desirous of receiving practical information that could be directly translated into their daily nutrition-related behaviours. When discussing their favourite aspects of the course or those they considered most useful, the most frequently mentioned elements were those relating to purchasing and preparing foods.

#### Knowing what to buy

Reflecting the large number of products available in the marketplace and the competing marketing messages to which consumers are exposed, participants often expressed considerable surprise when advised about the actual health attributes of various foods. The extent of the difference between their previous knowledge and the information provided was often substantial, and as such was perceived to be of considerable value:

‘Definitely new information for all participants – they were very vocal about being previously unaware of the level of sugar/fat/salt in food.’ (Observation field note)

‘You have no idea how valuable this has been to me. I have learnt so much today that my head is in a spin. I was surprised by the foods [variety of foods] that were healthy and could be eaten … even though I thought I knew, I thought I got it, I realised I didn’t.’ (Female, 50+ years, in-session survey)

The new information obtained from the course related to decisions both within and between food categories. Participants raised specific examples of: (i) changes they have made in terms of switching between brands of products once they became aware of substantial nutritional differences; (ii) making foods themselves rather than buying them because of the health and cost benefits; and (iii) changing product categories entirely to avoid unhealthy foods and replace them with healthier alternatives.

‘The whole course has helped me choose healthier food. I don’t buy ice-cream anymore, I eat more fruit and vegetables and multigrain bread.’ (Female, in-session survey)

Some participants noted that they appreciated being informed of new foods that are nutritious and easy to prepare and incorporate in familiar dishes. Chickpeas, lentils and couscous were nominated as unfamiliar foods that they now understood to be positive additions to their diets:

‘Her favourite part was being introduced to new foods like beans and lentils.’ (Observation field note)

‘I have never had couscous before, but I am going to use it now.’ (Female, 50+ years, in-session survey)

#### Knowing how to buy

Along with specific examples of products that represent sound nutritional choices and good value for money, the participants were especially appreciative of the product selection skills they were taught. The main skill mentioned was the ability to interpret food labels, followed by the ability to allocate foods to the Healthy Eating Pyramid, assess foods based on the ‘price per kilo’ method, and identify negative nutrients according to their various technical and common language terms. These skills provided participants with a means of continuing their nutrition education on an ongoing basis:

‘It left us consciously aware of looking at labelling instead of just walking straight up to the shelf and seeing what you want and taking it without looking at the label.’ (Male, Aboriginal focus group)

‘I definitely look at the cost of food differently. You look at some heavily processed snack food and you think, “That’s just a couple of dollars”, but when you break it down to per kilo, the amount of fruit and veg that you can buy for the same per kilo price is a lot more. So I definitely look at that more, and when I kind of look at some things and go, “Oh gee, they’re expensive today!”, but they’re still cheaper per kilo than something else. So I’m not hesitating to buy some of the fruit and veg when I’ve thought it was perhaps a little bit more expensive than I would have liked.’ (Female, mothers focus group)

‘She said she had learned different words for sugars, fats, and salts and that she will now look for these things in ingredients lists when shopping.’ (Observation field note)

Other skills valued by participants included memorable mnemonics and the utility of buying in bulk. An example of a mnemonic that was considered particularly useful was an image of a shopping trolley divided into three sections according to food categories. The baby seat was allocated to foods that should be consumed infrequently; the space under the baby seat was for meat and dairy products; while the rest of the trolley was assigned to plant foods:

‘The shopping trolley idea – it’s interesting because we usually fill our trolley by just chucking things in. This is a conscious way of shopping.’ (Female, in-session survey)

‘One Aboriginal lady said she [first] did this course a year ago and it has helped her to make a lot of changes to her family’s diet since completing the course … She now buys food in bulk to capitalise on specials.’ (Observation field note)

#### Knowing how to prepare

Participants were interested in learning how to make tasty foods that are also healthy and inexpensive. The most salient aspects of food preparation appeared to be the incorporation of more vegetables into meals and the use of substitutions to make their own recipes healthier:

‘He really liked making quiche because he hasn’t made it before and it was a new experience. He said he didn’t realise how easy and simple it was to make.’ (Observation field note).

Female 1: ‘I’ve definitely changed my cooking. So even if I’m cooking a recipe that I would normally cook that used cream, I’ve been using substitutes.’

Female 2: ‘And less salt. When I would be cooking something with tomatoes in it I used to go straight for the salt, and now I think, “No salt”. If anyone wants salt, then they can put it on after. Not to always rely on salt in cooking flavour.’ (Females, community focus group).

Other food preparation content areas that were mentioned as being useful by participants related to hygiene, specifically correct hand-washing and food storage techniques:

‘I got a lot from the washing hands exercise. I have always been a bit hit and miss with washing my hands. I now take better care to wash more thoroughly.’ (Female, online survey)

### Experiential aspects

Although the desire for and achievement of specific functional outcomes dominated participants’ responses, experiential aspects were also important. There was a strong preference for active involvement, especially in the form of cooking. Participants reported that engaging in food preparation during the sessions was a highly enjoyable activity that gave them the confidence and ability to prepare healthy meals at home, while also providing the opportunity to taste the prepared meals to ensure they were palatable. Similarly, learning activities that involved viewing real product examples, manipulating ingredients (e.g. measuring out the number of spoonfuls of sugar in sugar-sweetened beverages) and working with tangible stimuli (e.g. their own shopping receipts) were described as being both educational and enjoyable:

‘I found it was quite well thought out. Like when she came with props and all the visual aids. It wasn’t just someone talking to us; it was actually getting us involved with the activities. I found that kept you interested and motivated.’ (Female, community focus group).

‘The sugar in drinks activity was especially interesting and shocking to the ladies.’ (Observation field note)

The final experiential aspect of note was the interaction that occurred in the form of questions asked of the course instructors by participants:

‘Very informative, being able to ask questions. Friendly atmosphere which encouraged people to participate.’ (Female 50+ years, in-session survey)

The importance of the experiential style of the course was most apparent in the topics listed as ‘least preferred’ aspects of FOODcents. Few participants listed any negatives when given the opportunity, but those who did occasionally mentioned a preference for more cooking sessions and more time to ask questions.

### Social context

When asked about their favourite aspects of the FOODcents course, some participants explicitly mentioned that favourable interactions with other people were an important and enjoyable part of the learning experience:

‘It’s nice cooking with others and pleasant talking.’ (Migrant female, in-session survey)

‘Fellowship. Exchanging thoughts and ideas.’ (Male, 50+ years, in-session survey)

‘The getting together, cooking, company. It was fun.’ (Male, 50+ years, online survey)

The importance of social connections to the way the course was experienced and evaluated was also apparent in spontaneous mentions of how participants’ learnings could be used to benefit other people:

‘Very useful with the grandchildren when over, and husband. Thank you very much.’ (Female, 50+ years, online survey)

‘One lady was particularly interested in activities and took photos with her phone to show friends and family members.’ (Observation field note)

A further manifestation of the importance of the social aspects of the course was the reported tendency for participants to communicate with family and friends to espouse the benefits of FOODcents. Positive word-of-mouth behaviours occurred at two levels: programme and programme content. The former involved advising others of the existence of FOODcents, explaining its value and encouraging participation. The latter involved passing on specific pieces of information or recipes to promote healthy eating behaviours among others:

‘I’ve definitely talked about it to people.’ (Female, mothers focus group)

‘I told my children and grandchildren. I told them to read that [recipe] booklet.’ (Female, community focus group)

## Discussion

Previous programme evaluation research has demonstrated the potential for face-to-face adult nutrition education to achieve positive results in terms of nutrition knowledge and behavioural change^(^ ^4^ ^,^ ^6^ ^)^. To complement this work, research that focuses on the most rewarding aspects of the nutrition education experience for disadvantaged participants can inform future recruitment efforts to encourage those who need them most take advantage of programmes on offer. As participants’ perceptions of nutrition education programmes influence the extent to which they will act on the information provided^(^ ^23^ ^)^, this approach is also useful to guide ongoing programme refinement to enhance effectiveness. The present study builds on previous quantitative evaluations of the Western Australian FOODcents programme^(^ ^4^ ^,^ ^6^ ^)^ by providing an analysis of extensive qualitative data relating to participants’ experiences.

As outlined in the framework shown in Table 1, the attributes most appreciated by FOODcents participants were well aligned with the stated objectives of the programme^(^ ^5^ ^,^ ^18^ ^,^ ^24^ ^)^. Participants sought concrete, functional learning outcomes to facilitate improvements in their own and their families’ diets in a cost-effective manner. Participants especially valued specific product recommendations and the teaching of skills relating to interpreting food labelling and using ingredient substitutes in their favourite recipes. Also of importance was the ease of application of the information provided and assurance of the palatability of the recommended dietary changes. These findings are consistent with those of previous research that has focused on specific population sub-segments and identified a desire for user-friendly advice and skills that can be readily implemented at the point of purchase and in the home^(^ ^19^ ^,^ ^25^ ^)^. The present study provides further evidence that these course attributes should be a primary focus of messages used to recruit disadvantaged individuals to nutrition education programmes.

Second to a desire for functional outcomes, and related to achieving this goal, was a stated preference for an experiential style of learning that includes actively engaging in activities that are directly relevant to the subject matter. The adult education literature emphasises the importance of physically interacting with new information to achieve learning^(^ ^15^ ^–^ ^17^ ^)^. In the context of nutrition education, the inclusion of a cooking component has been noted to increase confidence and enjoyment^(^ ^19^ ^,^ ^25^ ^)^. The present results contribute to the small but growing body of evidence supporting the efficacy of this approach to nutrition education. Such evidence is important given the high per capita costs associated with delivering this kind of intervention and hence the need to demonstrate the potential benefits. In addition, the results emphasise the utility of other ‘hands-on’ activities that can enhance understanding of nutrition concepts among low-literate participants who may struggle to access nutrition information via other means. The ability to interact with the instructor throughout the course was considered by participants to be a further advantage that enhanced their learning experience.

Finally, various forms of social interaction were identified as being relevant to individuals’ experiences while attending FOODcents courses. Participants discussed the importance of being with other people who were in need of nutrition education during the sessions, and also indicated that they were constantly reviewing the information provided in terms of how it could benefit family members and friends. Such benefits included being able to: (i) prepare healthy, tasty foods for others; (ii) share their new knowledge to enable others to take advantage of the information and skills learned; and (iii) advocate about the course to others who could also benefit from attendance. The identified social aspects were therefore more extensive than has been typically recognised in adult nutrition education research, with previous studies focusing predominantly on the advantages of the group setting for information dissemination and skills teaching^(^ ^25^ ^,^ ^26^ ^)^. This finding relating to the importance of various social aspects of course attendance suggests that testimonial-style promotional messages may be particularly effective in promoting nutrition education programmes.

A strength of the present study was the extensive body of qualitative data that permitted the development of a proposed conceptual framework of the various aspects of nutrition education programmes that were especially appreciated by participants. Inevitably, the use of a qualitative approach precludes statistical analysis, limiting the generalisability of the findings. In addition, the Western Australian context of the study may have resulted in idiosyncratic outcomes. However, the consistency of the results with previous qualitative evaluation studies undertaken with different population sub-segments provides assurance of the trustworthiness of the findings. Further validation research is required across a broad range of contexts to assess the extent to which the results are generalisable to other groups of disadvantaged adults. A further limitation is the reliance on data obtained from participants who completed a FOODcents course. Those who enrolled but failed to complete the course may have had different experiences and a future focus on reasons for attrition could provide valuable information for further improvement of nutrition education programmes^(^ ^26^ ^)^.

## Conclusion

To conclude, the conceptual framework suggested by the data generated in the present study provides a guide for those developing or refining nutrition education programmes targeting disadvantaged people. Incorporating the aspects of most importance to participants into programme delivery and promotional messages may increase joining rates, enjoyment, satisfaction with course content and, ultimately, the uptake of recommended behaviours^(^ ^14^ ^,^ ^23^ ^)^. This outcome would be highly desirable to reduce current inequalities in nutrition knowledge and nutrition-related health problems between individuals of differing socio-economic status.
